# Association of type 1 stiff-person syndrome and insulinoma: a case report and narrative review

**DOI:** 10.1007/s42000-025-00666-y

**Published:** 2025-05-13

**Authors:** Michela Sibilla, Guglielmo Beccuti, Sara Belcastro, Umberto Mortara, Simone Parisi, Donata Campra, Alessandro Piovesan, Bruno Ferrero, Mauro Maccario, Mauro Papotti, Alessandro Maria Berton

**Affiliations:** 1 Division of Endocrinology, Diabetes and Metabolism, Department of Medical Sciences, University of Turin, Corso Dogliotti 14, 10126 Torino, TO Italy; 2 Division of Pathology, Department of Oncology, University of Turin, Turin, Italy; 3 Division of Rheumatology, Department of General and Specialist Medicine, Città Della Salute E Della Scienza University Hospital, Turin, Italy; 4 Division of General and Emergency Surgery, Department of General and Specialist Medicine, Città Della Salute E Della Scienza University Hospital, Turin, Italy; 5 Division of Oncologic Endocrinology, Department of Oncology, University of Turin, Turin, Italy; 6 Division of Neurology, Department of Neurosciences, University of Turin, Turin, Italy

**Keywords:** SPS, Paraneoplastic, Neuroendocrine tumor, Autoimmune disease

## Abstract

**Introduction:**

Stiff-person syndrome (SPS) is a rare neurological disorder that causes progressive muscle rigidity, gait disturbances, and functional impairment; type 1 is autoimmune, with positive anti-GAD antibodies (Ab), while type 2 is paraneoplastic and associated with antiamphiphysin Ab.

**Case presentation:**

A 41-year-old man with a silent medical history presented with stiffness and functional impairment; after numerous rheumatological and neurological investigations, he was diagnosed with SPS, with evidence of high titer anti-GAD Ab. After treatment with benzodiazepines was started, the patient began to experience episodes of confusion, which persisted even after reducing the dosage. During one of these episodes, he was admitted to the emergency department and a glucose level of 26 mg/dL was found. Differential diagnosis led to detection of an insulin-secreting neuroendocrine tumor of the pancreas; thus, a paraneoplastic origin of SPS was hypothesized. However, antiamphiphysin Ab were negative, anti-GAD Ab were persistently elevated, and symptoms only transiently improved after removal of the tumor.

**Conclusion:**

This is the first case, to our knowledge, demonstrating association between type 1 SPS and insulinoma, along with describing partial and transient improvement of neurological symptoms after resolution of the associated hypoglycemic syndrome.

## Introduction

Stiff-person syndrome (SPS) is a central nervous system disorder which was first described in 1956. It is characterized by progressive muscle rigidity (usually starting from the trunk and subsequently affecting distal limb muscles) and painful spasms that lead to gait difficulties, frequent falls, and a “wooden-man” appearance [1]. SPS has an estimated prevalence of 1–2 cases per million and an incidence of one case per million per year, with most patients presenting between the ages of 20 and 50 [2]. When initially described, little was known about the pathophysiology of the disease, which was initially thought to be a psychiatric disorder; it is now established that SPS is an autoimmune disease associated with different types of antibodies (Ab), even though the pathogenesis is yet to be fully understood [1]. Currently, therapeutic options for SPS include benzodiazepines, levetiracetam or pregabalin, oral/intrathecal baclofen, rituximab or tacrolimus, intravenous immunoglobulins, or plasmapheresis [3].

The prognosis is variable and depends on the initial presentation. Unfortunately, however, many patients remain symptomatic despite the availability of multiple therapies, their quality of life is severely affected, and depression is a frequent comorbidity [4].

**Fig. 1:**
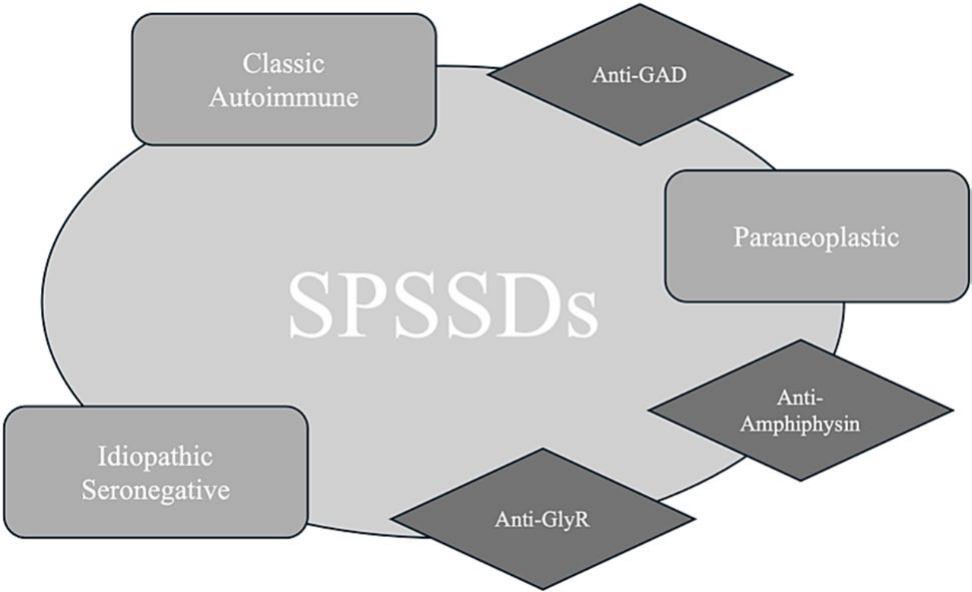
Antibodies in stiff-person syndrome spectrum disorders (SPSD) and most common clinical subtypes (GAD: glutamic acid decarboxylase; GlyR: glycine receptor)

From a pathogenetic point of view, SPS identifies the following three different patterns: type 1 (associated with other autoimmune conditions and typically linked to antiglutamic acid decarboxylase-GAD-Ab), type 2 (paraneoplastic, mainly associated with antiamphiphysin Ab), and type 3 (idiopathic, seronegative) [3, 5] (Fig. 1).

Type 1 is the most common form, with Ab reacting with the 65 kDa isoform of GAD found in 60–80% of cases [6]. As already mentioned, SPS may be associated with several autoimmune disorders (e.g., pernicious anemia, vitiligo, celiac disease, and myasthenia gravis), including endocrine diseases, such as type 1 diabetes mellitus (T1DM), Hashimoto’s thyroiditis, and Graves’ disease [1, 7]. In addition, an association with latent autoimmune diabetes of the adult (LADA) has also been described [8].

The association with T1DM is the most common as the two disorders share anti-GAD Ab; T1DM is in fact found in about 30% of SPS patients, but only one in 10,000 patients with T1DM is affected by SPS [9]. Of note, anti-GAD Ab are found in both blood and cerebrospinal fluid of SPS patients, but occur only in the blood of T1DM patients [1]. Several theories have been proposed to explain the difference in pathogenicity: it has been suggested that anti-GAD Ab recognize different epitopes of the same molecule in the two conditions [10], or else the difference could be related to the higher titer of Ab present in SPS compared to T1DM [11].

SPS type 2 represents less than 10% of cases and usually presents in women with breast cancer [12].

The association of SPS and cancer has been known for years and several hematological as well as solid malignancies have been reported, including endocrine tumors [13]. A recent case report described a patient with atypical carcinoid of the lung and paraneoplastic SPS secondary to antiamphiphysin Ab [14].

To our knowledge, however, the association between SPS and hormonally active pancreatic tumors, including insulin-producing neuroendocrine tumor (NET), has never been reported.

We hereby present the case of a 41-year-old man with a GAD-related SPS who developed an insulinoma 1 year after SPS diagnosis.

## Case presentation

The patient is a 41-year-old male with no known allergies and a silent medical history, except for an appendectomy at the age of 20.

In July 2021, after the second dose of the Spikevax vaccine, he began to experience muscle rigidity with functional impairment.

He subsequently underwent a series of rheumatological and neurological investigations (Table 1) that did not yield a definitive diagnosis. From the beginning, the diagnosis proved difficult due to the non-specific nature of the symptoms and the fact that they never fully met the classification/diagnostic criteria of the conditions. Initially, he was diagnosed with myositis, followed by a diagnosis of spondyloarthritis. Treatment for the latter condition included methotrexate, prednisone, celecoxib, and etanercept; however, there was no clinical improvement. The patient discontinued treatment due to episodes of confusion.

**Table 1:** Rheumatological and neurological investigations undergone by the patient

Exam	Results
ANA screening	1:640 positivity High titer of anti-SSA (Ro60)
Myositis specific antibodies	Anti-OJ (anti-isoleucyl-tRNA synthetase), that subsequently became undetectable after surgery
Muscle biopsy	CD4 + and CD8 + lymphocyte (interpreted as antisynthetase antibody myositis)
Four-limb muscle motor evoked potential	Negative
Encephalic MRI and angio-MRI	No significant findings

*ANA* antinuclear, *SSA/Ro* Sjogren’s syndrome-related antigen A autoantibodies, *CD* cluster of differentiation, *MRI* magnetic resonance imaging

Finally, in March 2023, after an electromyography (EMG) control test, which showed a typical pattern with continuous motor unit activity in agonist and antagonist muscles, and the detection of anti-GAD circulating Ab, a diagnosis of SPS was established based on the diagnostic criteria summarized in Table 2 [15]. The diagnostic criteria for this disease, however, are still a matter of debate in the literature [16]. Treatment with clonazepam at the initial dose of 5 drops (0.5 mg) *bid* was started, with partial improvement of symptoms. When the dosage was increased to 5 drops *tid*, the patient reported daytime drowsiness and excessive tiredness. An attempt was made to reduce the morning dose, increasing the bedtime dose, but the periods of “confusion” and spatial–temporal disorientation persisted (exacerbated by events of intense psychophysical stress) and the patient had to be admitted to the ER for these symptoms several times.

**Table 2:** Stiff-person syndrome diagnostic criteria [15]

1.Clinical symptoms (stiffness or episodic spasms)
2.Clinical signs during symptomatic phase of illness (increased muscle tone or exaggerated lumbar lordosis or concurrent stiffness of lumbar, paraspinal and, abdominal muscles)
3.Serological findings (evidence of GAD65 IgG or glycine-R IgG or amphiphysin IgG)
4.Electrophysiological studies (inability to relax paraspinal muscles or exaggerated acoustic/exteroceptive responses by surface EMG or co-contraction of agonist and antagonist muscles)
5.Exclusion of alternative diagnosis

Definite SPS (all), probable SPS (1 or 2, 3, and 5) or (1, 2, 4, and 5) *GAD* glutamic acid decarboxylase, *IgG* immunoglobulin G, glycine-R: glycine receptor, *EMG* electromyography. *This table is an adaptation from the original article*

In June 2023, the patient was readmitted to the ER due to confusion and disorientation in space and time. Blood exams showed a glucose value of 26 mg/dL with no other relevant findings. The patient was therefore administered two vials of 33% glucose solution (GS) with subsequent infusion of 10% GS at 100 mL/h. Blood glucose was restored to normal values and a regression of symptoms was observed. A cranial CT scan was performed, but no acute ischemic or hemorrhagic lesions were detected. An abdomen ultrasound revealed no anomalies, showing normal pancreatic structure and texture. He was then seen by an endocrinologist and hospitalized in the City of Health and Science Hospital of Turin, Italy, on suspicion of a hypoglycemic syndrome of unknown origin.

Despite continuous infusion of 5% GS and frequent fractionated meals, the patient had hypoglycemia symptoms on several occasions; during one such episode, a blood glucose of 58 mg/dL was recorded associated with insulin and c-peptide levels of 4.8 mIU/L and 2.2 mcg/L, respectively.

In the following days, the only regimen that allowed maintenance of normal glucose levels was the infusion of 5% GS during the day and 10% GS at night (with mean glucose values of 80 mg/dL).

After a careful medical history review, a low fasting blood glucose level was observed in exams during May 2022 (53 mg/dL) and an episode of confusion relieved by the intake of sugars was reported in January 2023, followed by several unexplained similar events in the following months.

### Differential diagnosis

The main diagnostic hypotheses at this point included the following: T1DM onset (as the association between the two diseases is well established and the patient had anti-GAD Ab), Hirata syndrome (even though the patient denied any use of supplements containing alpha-lipoic acid), and insulinoma (as insulin and C-peptide were not suppressed during an episode of moderate hypoglycemia). Therefore, a subsequent biochemical evaluation including a complete panel of Ab (Table 3), was requested and, on this basis, both T1DM and Hirata syndrome were excluded. Severe hypothyroidism and adrenal insufficiency were ruled out as well.

**Table 3:** Biochemical evaluations performed during the hospital stay and follow-up

Diagnosis	Parameter	Values at diagnosis	Values at 6 months
T1DM	Anti-insulin receptor	Negative	Negative
	Antipancreatic islet cells (UJDF)	Positive	Positive: 40
	Antityrosine phosphatase	Negative	Negative
	Antizinc transporter	Negative	/
	HbA1c (mmol/mol)	17	33
Hyrata syndrome	Anti-insulin	Negative	Negative
SPS	Anti-GAD (U/mL)	Positive: > 250	Positive: 1110
	Antiamphiphysin antibodies	Negative	Negative
Atrophic gastritis	Antiparietal cells	Negative	Negative
	Anti-intrinsic factor	Negative	Negative
Celiac disease	Antiendomysium	Negative	Negative
Thyroiditis	Anti-TPO (kIU/L)	Positive: 14.9	Negative
	TSH (mIU/L)	1.032	1.522
	fT4 (ng/L)	8	10
Adrenal insufficiency	Morning serum cortisol (mcg/L)	160	224
MEN1	Calcium (mg/dL)	9.6	9.7
	PTH (pg/mL)	14.3	28.8

*T1DM* type 1 diabetes mellitus, *HbA1c* glycated hemoglobin, *SPS* stiff-person syndrome, *GAD* glutamic acid decarboxylase, *TPO* thyroid peroxidase, *TSH* thyroid-stimulating hormone, *fT4* free T4, *MEN1* multiple endocrine neoplasia type 1, *PTH* parathyroid hormone

No further autoantibodies emerged from the screening of other possible autoimmune diseases, except for AbTPO and ICA detected at higher titers.

Consequently, a brief fasting test (Table 4) was performed, leading to the diagnosis of endogenous hyperinsulinism. A therapy with diazoxide was started at the dose of 25 mg *tid* and progressively increased up to 100 mg *qid*, with maintenance of 10% GS only at night.

**Table 4:** Brief fasting test, with glucose measurements every hour, which led to endogenous hyperinsulinism diagnosis

	10:00 am	11:00 am	12:00 pm	13:00 pm
Blood glucose (mg/dL)	44	37	35	30
Insulin (mIU/L)	6.7	5.7	6.2	5.3
C-peptide (µg/L)	2.2	2.0	2.0	2.0

After a negative contrast abdomen and thorax CT, an echoendoscopy was performed showing a hypovascularized and heterogeneous tumor measuring 17 × 18 mm in the tail of the pancreas, which was highly suspicious of a neuroendocrine tumor. The fine needle aspiration material confirmed this suspicion in the presence of medium-sized neoplastic cells positive for chromogranin A, consistent with a well-differentiated neuroendocrine tumor.

### Surgical treatment

The patient underwent a laparoscopic distal pancreatectomy, with no periprocedural complications. He was then discharged with the indication to continue daily glucose monitoring but with no need for a specific endocrinological therapy.

### Histopathology examination

**Fig. 2:**
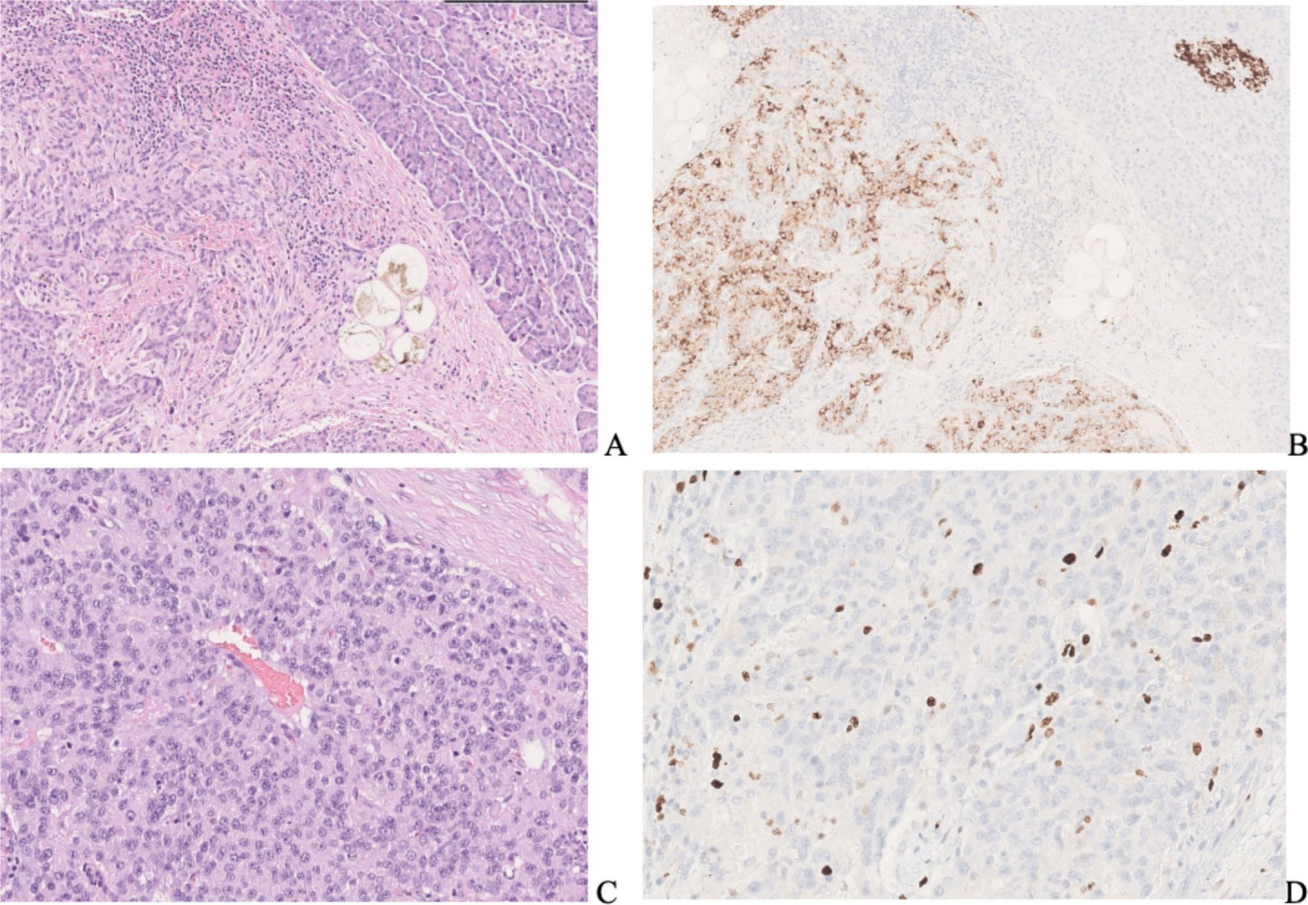
Well-differentiated tumor of the pancreas with an organoid growth (**A**) producing insulin in most neoplastic cells (**B**) (top right: a control islet of Langherans contains insulin positive cells). The tumor is composed of medium-sized cells with minimal atypia (**C**) and, in the presented case, had a low Ki67 proliferative index of approximately 5% in hot spot areas (**D**)

A trabecular and solid growth was observed composed of relatively homogeneous round cells producing chromogranin A and insulin, supporting the diagnosis of a well-differentiated insulin-producing neuroendocrine pancreatic tumor (Pan-NET) with free resection margins. According to the WHO 2022 classification, the tumor was grade G2 based on a mitotic count of 2 mitoses per 2 square mm and a Ki67 proliferative index of 8% in the highest labeled areas (Fig. 2). The pathological UICC staging (VIII edition) was pT1.

### Outcome and follow-up

After surgery, no more hypoglycemic events occurred and glucose monitoring could be discontinued. Biochemical evaluation showed normal glucose, insulin, and chromogranin A (33 ng/mL) levels.

From the neurological point of view, the patient gradually reinitiated physical activity and was able to do movements that were previously very difficult due to rigidity, such as shoe-lacing and lower limbs flexion on the trunk. He was also able to go on holiday and swim and gradually increase the intensity of the physical activity. The dosage of clonazepam was reduced to 5 drops (0.5 mg) in the morning and 10 (1 mg) at night. Unfortunately, after a few months, the patient reported an exacerbation of symptoms.

Currently, 12 months after surgery, there is no evidence of reoccurrence of the pancreatic lesion nor hypoglycemia episodes, but the patient’s neurological symptoms persist.

## Case discussion and literature review

To our knowledge, we herein describe the first confirmed case of an association between SPS and insulinoma with almost simultaneous occurrence of symptoms related to the two conditions. Moreover, our case is even more peculiar considering that he was simultaneously harboring both high titers of anti-GAD Ab (suggestive of type 1 SPS) and a possible paraneoplastic syndrome (more common in SPS type 2).

GAD is the rate-limiting step in the decarboxylation of L-glutamate to γ-aminobutyric acid (GABA). Two isoforms of GAD exist, namely, a cytoplasmic constitutively active form (GAD67) and a synaptic membrane-associated form (GAD65), the former providing a steady production of GABA and the latter supplying pulses of the neurotransmitter when post-synaptic inhibition is needed [1].

As mentioned above, although patients with SPS and T1DM both have high levels of anti-GAD65 Ab, these immunoglobulins might recognize different epitopes of GAD65 in the two diseases [10, 17]. Other authors, on the other hand, propose that the difference in pathogenicity is related to the higher titer of anti-GAD Ab in SPS rather than differences in epitope recognition [11].

SPS is associated not only with T1DM but also with other autoimmune diseases, namely, endocrinological diseases.

A retrospective study conducted in Taiwan [7] examined 14 cases of SPS and their association with autoimmune disorders.

All patients who tested positive for anti-GAD Ab had classical SPS; in addition, two of them had autoimmune thyroiditis and were positive for anti-TPO Ab, one had T1DM, and one had LADA. Interestingly, though the two patients who had an autoimmune form of diabetes had low titers of anti-GAD Ab (100–1000 U/mL) when the disease was diagnosed, the levels rose to > 2000 IU/mL when they developed SPS. This could confirm the hypothesis that higher titers of anti-GAD Ab are needed for SPS to manifest.

Of the six patients who were negative for anti-GAD Ab none had autoimmune diabetes or thyroiditis; however, one had Sjögren’s syndrome (ANA positive and anti-Ro antibody positive), one had myasthenia gravis (Ach-R Ab positive), and two had neoplasms (one lung cancer with paraneoplastic syndrome and one thymoma).

In addition to T1DM, LADA, and autoimmune thyroiditis, 5–10% of patients with SPS also have Graves’ disease, pernicious anemia, or vitiligo [6]. Moreover, SPS is also associated with autoimmune polyglandular syndrome type 1 (APS1) and type 2 (APS2). APS1 includes mucocutaneous candidiasis, hypoparathyroidism, and Addison’s disease but is less frequently associated with T1DM, Hashimoto’s thyroiditis, or chronic hepatitis. APS2 consists of Addison’s disease plus either autoimmune thyroiditis or T1DM and is associated with hypogonadism, pernicious anemia, celiac disease, and primary biliary cirrhosis [18].

Intriguingly, Yin Lee et al. [19] recently reported a case of SPS induced by exogenous insulin injection in a patient with LADA. He had positive anti-GAD and anti-insulin Ab and an insulin deficiency, compatible with the diagnosis of LADA. He began to experience rigidity after insulin was introduced to such a point that he discontinued treatment and had poor glycemic control for over 10 years. When insulin was added again, rigidity worsened: a tolerable abdominal rigidity was finally achieved by splitting the administration of basal insulin into two separate injections 12 h apart.

Our patient did not receive exogenous insulin but was exposed to inappropriately high levels of endogenous insulin for several months. Although further evidence is needed to establish a definitive association between SPS and the immunological response to insulin, the potential involvement of insulin or insulin Ab in the pathogenesis of SPS cannot be ruled out and warrants further investigation.

A stiff-person-like syndrome has also been described in cases of adrenal insufficiency [20]; in such cases, it is postulated that the pathophysiology revolves around hyponatremia and glucocorticoid deficiency which lead to reduced activity of the Na–K-ATPase and eliminate beta-adrenergic stimulation to the Na–K pump. However, it is important to differentiate rigidity induced by SPS from that induced by cortisol deficiency. In the first case, rigidity involves both flexion and extension and limbs are held in extension; in the latter, flexion deformities are predominant. In our patient, however, hypocortisolism was excluded as a concomitant cause of the disorder.

Of note, our patient began to experience SPS symptoms after the second dose of the Spikevax vaccine, although only the Sars-Cov- 2 infection itself has so far been reported in association with SPS. In one case, a patient developed a transient and reversible stiff-person-like syndrome during the viral infection [21], while another patient, who was already affected by stiff-limb-syndrome, experienced a worsening of the syndrome after the infection, with the development of generalized SPS [22]. Notably, several cases of T1DM with positive GAD Ab triggered or worsened by Covid- 19 have been reported which were probably due to augmented proinflammatory cytokines and recruitment of CD8 + T cells [23].

As previously mentioned, three subtypes of SPS are currently recognized, with type 2 amphiphysin-positive SPS being the paraneoplastic form [3, 5]. Recently, the broader term SPS spectrum disorders (SPSSDs) has been introduced into clinical practice to include a series of diseases with signs and symptoms similar to those of SPS [24].

Breast cancer is the most common carcinoma linked to SPSSDs, these patients being found to suffer from other diseases, including autoimmune disorders such as paraneoplastic encephalomyelitis, T1DM, thyroid disease, rheumatoid arthritis, and sarcoidosis. The main antigens found in the latter patients are amphiphysin, followed by GAD65, acetylcholine receptor, and glycine receptor (GlyR). Conversely, in lung cancer associated with SPSSDs, the most common antigen is GAD65, followed by amphiphysin. In hematological cancers such as lymphoma, on the other hand, GlyR is the most frequently found antigen. SPSSD is also associated with other tumors such as pancreatic, colorectal, renal cell, embryonal, ovarian and prostate carcinomas, testicular seminoma, multiple myeloma, glioma, melanoma, and liposarcoma [13].

In terms of pathogenesis, the GAD antibody inhibits GAD65 from blocking GABA synthesis, thereby reducing the uptake of newly synthesized GABA in synaptic vesicles and its synaptic release [25]. This induces decreased GABAergic transmission, which leads to neuronal hyperexcitability and is the core pathophysiological mechanism in SPS [26].

Amphiphysins are members of the Bin-Amphiphysin-Rvsp (BAR) proteins; amphiphysin I is expressed in mammalian brains and is associated with SPS and breast cancer, while amphiphysin II is associated with cancer progression and myopathies [27]. Type I and IIa share a brain-specific domain, while IIb has a skeletal muscle-specific domain with a tumor suppressor that interacts with the c-Myc oncoprotein [28].

Glycine is an inhibitory neurotransmitter which, together with its receptor, is crucial for central nervous system development [29]. Its biological function is dependent on specific transporters such as GlyT1 (present in glial cells), which also regulates glutamatergic neurotransmission through N-methyl-D-aspartate (NMDA) receptor, playing a role in both brain function and in diseases [30]. Glial cells modulate synaptic development in white matter via GlyRs [31].

Some reports of neuroendocrine tumors associated with SPS have been published in the literature, but in these cases, a paraneoplastic antiamphiphysin positive form was present [14].

The patient reported herein had positive anti-GAD65 Ab and negative antiamphiphysin Ab; although antiamphiphysins most frequently occur in paraneoplastic SPS, as described above, different tumors are associated with different autoantigens. Notably, Yohannan et al. [32] also reported a case of a 20-year old patient affected by mediastinal liposarcoma and SPS who harbored the GAD65 autoantibody but tested negative for antiamphiphysin; after resection of the primary mass, her overall performance status improved with outpatient physical therapy and she gained independence in her daily activities. However, the authors report that she still has some residual ataxia and remains on diazepam. Even in the oncological setting though, the prognosis of SPS is hard to predict. A previous case described a patient who developed SPS during a first relapse of Hodgkin’s disease, which completely regressed after chemotherapy treatment; when a second relapse of the hematological disease occurred, however, no signs of SPS were detected, indicating that SPS is a sporadic paraneoplastic manifestation with an unpredictable trend [33]. Nevertheless, SPS prognosis is generally unfavorable, with persisting symptoms despite multiple lines of treatment [4].

Hence, the clinical evolution of our patient, with a transient marked improvement of symptoms after resection of the tumor, is more likely due to the absence of constant hypoglycemic episodes rather than to a resolution of his neurological condition; a role of placebo effect after the surgery, with removal of the apparent cause of disease for the patient, must also be considered.

Our case report has some limitations. Firstly, anti-GlyR Ab, which could also potentially be associated with paraneoplastic SPS, were not determined given the unavailability of the assay in our laboratory. Secondly, a definite cause-effect relationship between SPS and the insulin-producing NE tumor cannot be assessed, nor can it be clearly established whether the patient had a type 1 SPS, a paraneoplastic SPS, or an overlapping form of the two.

## Conclusions

We have presented the first case, to the best of our knowledge, of confirmed association between SPS and insulinoma in a patient who had positive anti-GAD Ab and negative antiamphiphysin Ab. The onset of neurological symptoms after vaccination and only transient improvement of symptoms after tumor resection probably indicate an autoimmune pathogenesis of the syndrome (SPS type 1). In any case, the overlap of the neurological picture with the neuroglycopenic manifestations rendered both the differential diagnosis and clinical management challenging. This case summarizes effectively the complex nature of SPS and the frequent interaction of this syndrome with the endocrine system.

## Data Availability

Not applicable.
